# Stimulating co-production in healthcare quality improvement: raising the interest of health professionals to collaborate with patients

**DOI:** 10.3389/frhs.2026.1779684

**Published:** 2026-04-30

**Authors:** Mats Brommels

**Affiliations:** Medical Management Centre, Department of Learning, Informatics, Management and Ethics, Karolinska Institutet, Stockholm, Sweden

**Keywords:** behavioural change, co-production, patient participation, professional attitudes, quality improvement

## Abstract

There is ample evidence that patients contribute substantially to quality improvement, i.a. by offering perspectives and opportunities for improvement not necessarily noticed by professionals and managers and increasing the patient-centredness of improved care processes. The literature on co-producing quality improvement highlights mostly structural and organisational barriers to patient involvement. However, several studies report about health professionals being sceptical to patient co-production in general and the same attitude might prevent them from engaging in collaboration in quality improvement. The vast literature on professional practice change, including changes informed by new scientific evidence and required by clinical guidelines, show the value of interventions guided by theories from social psychology. In this paper advice is sought from those theories on how to increase the willingness of health professionals to co-produce QI with patients. Based on a synthesis of that analysis the following recommendations are formulated: Showing the benefits of patient involvement to professionals and how those resonate with their professional beliefs and goals will have a positive effect on their attitudes towards co-production. Engaging professional champions to lead QI will create social pressure and establish norms that will attract other professionals to participate. Enabling professionals to actively participate in designing the improvement processes will provide them with a sense of control of their working conditions that will increase their intrinsic motivation. Institutionalising co-produced quality improvement as organisational routines will show professionals that QI is an ordinary day-today activity that will enrich their clinical practice.

## 1 Introduction

There is a broad consensus about the benefits of patient—and largely, public—participation in the provision and development of health services. A UK policy document states that “…. Patients and the public offer a unique voice to service development, identifying required improvements and inefficiencies first-hand as ‘experts by experience’.” The document goes as far as to claim that patient involvement “must run through the full cycle of every quality improvement project, as an integral part of the fabric of the whole project.” This will include a say on the strategic direction of QI projects and the outcomes to be measured, as well as participation in project governance and communication (1). These requirements fit well with the NHS England definition of co-production: “Co-production is acknowledged as an approach where professionals share power and have an equal partnership with people to plan, design and evaluate together” (2). Even with less ambitious approaches to patient participation in QI the full acceptance of all stakeholders responsible for healthcare service provision of involving patients is required– that includes senior executives, clinical managers and health professionals.

There is empirical research that supports such a policy about patient participation in QI. As noted by Iroz et al. (3) patient engagement has contributed to the design of new clinical guidelines and practices. In addition to care improvement, patients and their families appreciate new insights into the healthcare system, that their advocacy rights are affirmed and that they are appreciated members of QI teams. Patients also bring new perspectives that will enhance innovation. The radicality of those depend on the phase in which patients are involved. Most important is patient participation in the action phase of the project. Less impact will be found in the evaluation phase, and the least in the problem identification phase (4). There are also direct positive effects of patient participation in QI like increased patient adherence to treatments, improved clinical outcomes and patient satisfaction, as well as service provider productivity, as shown in a recent scoping review analysing 17 studies in detail (5).

Despite these obvious benefits involving patients in QI co-production is seen as challenging. The degree of patient involvement might differ even in the same provider organisation. Some QI teams have patient representatives as regular members; others consult patient representatives at one single occasion or collect structured information from patients by interviews or by arranging workshops (6).

Iroz et al. (3) studied the experience of patients and their family partners of participating in a national QI collaborative. Whilst they were motivated by the connection to their care team, their sense of community and the possibility to take project ownership, their proposed changes to the QI process related mostly to meeting procedures, including how to handle sensitive information.

Bergerum et al. (7) returned to scrutinise the challenges of patient participation by studying two healthcare provider organisations, one in Sweden and one in the Netherlands. The authors point to the fundamental nature of healthcare as one main obstacle. They write: “Hospital organisations, emphasising knowledge and evidence domains, are characterised by operational-professional rather than patient-preference led management. Thus, PPI [patient and public involvement] adds aspects of influence and responsibility that are not clearly defined or understood, with limited knowledge about how it can be orchestrated.” Although managers interviewed acknowledged the experiential knowledge of patients in regard to their health, ill-health and care and the importance of utilising it, they admitted that they identified themselves as professionals and felt that recognising patients as experts could make them feel uncomfortable (7).

This sentiment seems to be echoed more broadly among health professionals. Carstensen et al. (8) found that studies on the nature of the engagement of professionals in quality improvement collaboratives (QIC) indicate that professionals are, in general, reluctant to engage in QICs when those appear as “bureaucratic intrusion” into their professional practice and not meeting their professional interests. In another study QI project team members were, by and large, happy with working with patients. They met, however, challenges in convincing clinical staff to join the project. Some physicians expressed concerns about the possibility of receiving critical remarks from patients about their care (9). Even when the intention is to collaborate with patients the relationship might not be equal. Le Grouw et al. (10) observed in their study on shared decision-making in a surgical setting that “… interactions remained unequal and were directed towards seeking consent instead of consensus.”

I was triggered to specifically look into the question of the attitudes towards QI co-production among health professionals by insights I gained in our studies of Recovery Colleges (RCs; learning activities for service users to support coping with disease, organised by peers). We found that professionals in mental health showed conflicting views and attitudes towards collaboration with persons with lived experience (PwLEs). Little interest and sometimes hostility towards PwLEs and RCs were expressed in our interviews (Al-Adili et al., forthcoming) (11). We also noticed that only psychiatrists who had been actively involved in RCs themselves and seen their positive benefits actively planned to change their clinical practice to be more recovery-oriented (12).

### In summary: structural, organisational and cultural barriers to patient co-production in QI

There is a rich literature on strategies to involve patients in QI. Many studies show the benefits of active patient participation. On the other hand, most of those also refer to obstacles to such co-production. Most patient participation is by representatives of patient organisations rather than actual patients in the provider organisation. Engaging those “outsiders” is a recruitment challenge and they might have problems to fit schedules with ongoing QI activities (structural barrier). Meeting procedures and policies around confidentiality are also mentioned as problems (organisational barrier). Finally, and probably most importantly, there is reluctance among health professionals to join co-production with patients as equals (cultural barrier).

The literature on interventions to promote professional practice change based on evidence and the implementation of those interventions is vast and growing. The seminal work of Grol and Grimshaw (13) has been one of the most influential. Their research group emphasised already in 2005 (14) that effective change strategies are best guided by social psychology theories.

The aim of this paper is to explore how those theories can inform activities intending to encourage health professionals to more actively seek cooperation with patients and their families in QI.

## 2 Overview of social psychology theories and theories of practice change

### 2.1 Social psychology theories

A dictionary definition presents social psychology as the study of the manner in which the personality, attitudes, motivations, and behaviour of the individual influence and are influenced by social groups. (https://www.merriam-webster.com/dictionary/social%20psychology). Three specific phenomena are of interest: individual phenomena cover attitudes, emotions, decision-making and behaviour; communication is an example of interpersonal phenomena; and relations and interaction between individuals are collective phenomena. In the following, social psychology theories focusing on attitudes and behaviour will be presented and their relevance discussed.

One of the most commonly applied social psychology theories is the theory of planned behaviour (TPB). Crano & Prislin (2006) stated: “A voluminous TPB-inspired literature testifies to the model's heuristic value” (15). It predicts how attitudes, subjective norms, and perceived behavioural control shape an individual's behavioural intentions. Beliefs of norms, capacity and control form attitudes and lead to subsequent behaviour (16). More specifically, attitudes are formed by beliefs about outcomes and consequences of a behaviour, norms are the result of social pressure to conform, and perceived behavioural control stems from the feeling of the ability to perform under the influence of external and internal factors affecting behaviour (17). Attitudes control intentions that trigger behaviour. Sheeran (18) has estimated that, on average, 28% of the variance in behaviour is accounted for by differences in intentions.

Crano & Prislin (15) reviewed empirical studies on attitude-behaviour consistency. They summarised that behaviour is guided by attitudes in two alternative processes—deliberate or automatic. Motivation and a possibility to communicate leads to conscious deliberation resulting in a considered behaviour, whereas accessibility triggers spontaneous behaviour. Strong attitudes remain stable whereas weak ones are affected by behaviour. However, strong attitudes may not be manifested behaviourally if that behaviour is in conflict with self-interest. In addition, only attitudes supported by in-group norms would predict behaviour (15).

An example from healthcare illustrates these theoretical predictions. Chinese physicians were surveyed on their attitudes to patient-centred communication and orientation to share power with patients in medical decisions using a revised patient–practitioner orientation scale. A multi-level ordinal logistic regression analysis verified a significant association between physicians' attitudes towards patient-centred communication and a higher intention to involve patients in shared decision-making (19).

Applying these observations to healthcare, what essentially forms attitudes are in-group norms, learned during socialisation into one's profession, and beliefs in outcomes that are derived from professional knowledge. Consequently, the professional identity seems to be important, and advice from social identity theory can be expected to be relevant.

According to social identity theory attitudes and attitude change are affected by (i) concerns with self and others, (ii) rewards or punishment, and (iii) a valid understanding of reality (20). Connor and Norman (21) highlight the importance of contexts that are manifested in three forms that affect attitudes. An individual has values, goals and emotions that contribute to personal development. Social relationships have an impact through persuasive messages, social media, and by cultures. Finally, the sociohistorical and sociopolitical context is influenced by unique events including the economic development. Social relationships and sociopolitical context mediate interventions to bring about attitude changes, and those changes predict the effect of those on behavioural change. The authors find that these mechanisms are supported by several theories. Empirical studies, however, have shown that the effects of attitudes and intentions on behaviour are of medium or small size (21).

### 2.2 Interventions to promote professional practice change

The Cochrane Effective Practice and Organisation of Care (EPOC) Review Group has listed ten interventions used to achieve professional practice change: Distribution of educational materials, Educational meetings, Local consensus processes, Educational outreach visits, Local opinion leaders, Patient-mediated interventions, Audit and feedback, Reminders, Marketing and Mass media. Those are of varying effectiveness. http://epoc.cochrane.org/sites/epoc.cochrane.org/files/uploads/datacollectionchecklist.pdf. 2002.

Johnson and May (22) performed a systematic review of systematic reviews on the effectiveness of interventions to promote professional practice change. They analysed studies that reported the outcomes of those interventions applying a theoretical framework often used in implementation research (normalisation process theory). That sociological framework covers organisational and technological conditions as well as and collective action and provides a complementary perspective to the individual ones of social psychology. In addition, applying such a theory will enable the demonstration of patterns providing tentative causal mechanisms related to the effectiveness of the interventions studied. Actually, the authors chose to present their summary findings as hypotheses.

Their analysis shows a difference between action-oriented strategies that report more successes in comparison to strategies focusing on persuasion rather than action. To those they were able to link EPOC interventions. The latter strategies are referred to as attempts to “reshape the attitudinal landscape” by indirect measures to affect behaviour, using marketing and mass media campaigns or proposing new forms for practice in consensus building processes. In contrast, the action-oriented strategies engage opinion leaders and the introduction of guidelines to demonstrate new intended practices as something to adopt (“normative restructuring”). This is amplified by “relational restructuring” by emphasising the expectations of an external reference group, enacted through Educational outreach, Reminders, Audit and Feedback.

These strategies combined codify rules about professional practice and behaviour. Complying with those rules will be a normal part of everyday work where professionals will follow the example of their peers (22).

To view QI and co-produced QI as “a normal part of everyday work” implies that those activities could benefit from being managed as organisational routines. The change of routines is promoted by shared team leadership (23). If normative and relational restructuring affect professional attitudes and behaviours and support the establishment of a culture of co-produced QI, shared leadership could be argued to support in maintaining and further developing that organisational routine.

## 3 Analysis and discussion

The starting point for this paper was the notion that many health professionals feel reluctant to engage in co-production with patients, including quality improvement. The goal is to achieve a change in that behaviour and create conditions for a fruitful continuous professional-patient collaboration. How can such an initiative be guided by social psychology theories and the evidence of successful professional practice change?

Behavioural change follows supporting norms, beliefs in personal capacity, beliefs of control, meaning the ability to perform according to goals, and beliefs about the outcomes and consequences of the changed behaviour. There is convincing evidence of the benefits, also in terms of medical outcomes, of active patient participation in care planning, care interventions and follow-up even in severe diseases (24). Highlighting those benefits among health professionals is important, but it seems that is not a sufficient to change the minds of sceptical professionals.

Social psychology theories show that positive attitudes create intentions leading to behavioural change, although, as was noted above the effect might be only medium or small size. A more comprehensive strategy than attempting at changing attitudes by persuasion is needed.

Starting with the first step in the causal chain leading to change in behaviour, which is making patient co-production into a norm, will require active interventions by opinion leaders within each of the professional groups. In-group norms and peer pressure will have an effect on attitude and intention. The professional identity is formed during education and amplified during the professional career. Most fortunately, QI is already a topic in health professions education and defined as a core competency in many national residency programmes of clinical medicine (25). (It is notable that organised training in co-production is still very scarce, although some examples have been reported in the literature) (26, 27). Following the recommendations of Johnson and May (22) QI could be included in clinical guidelines and be part of regular clinical audit and feedback programmes. Such initiatives would also codify QI and co-production supporting norms and affect the “individual context” of personal goals and values, as emphasised by social identity theory. It would also create a supportive professional culture as a part of the social relationship context. One could expect that the growing focus on patient-centredness and patients' rights already has been impacting the sociopolitical context in healthcare in a way which promotes patient co-production.

Although group norms are anticipated to create more positive attitudes and thus intrinsic motivation to engage in co-production, social identity theory also points the issue of extrinsic motivation factors and possible conflicts with self-interest. QI is included in many performance management systems on the organisational level, and existing individual pay-for-performance arrangements could be used to the same purpose. The most important component of matching self-interest in a professional service is probably working conditions supporting the execution of professional tasks. Ensuring that co-produced QI safeguards good working conditions for all staff would minimise the risk of conflict with the self-interest of health professionals.

These proposed actions would constitute an action-oriented intervention to achieve professional practice change, and as argued by Johnson and May (22), create favourable conditions for a professional culture and practice engaging professionals in co-produced quality improvement. Such an action programme highlights the need for engaged leadership. Kjellström et al. (28) reviewed the literature on leadership of co-production and identified as essential practices, i.a., power-sharing, training, supporting, establishing trust and communicating, all matching central principles of co-production.

## 4 Conclusions

Highlighting the benefits of patient involvement to professionals will resonate with their professional goals and supposedly create a positive attitude to co-production, generally, and to QI. Engaging professional champions to lead QI will create social pressure and establish norms, and, as a consequence, incentives for professionals to participate. Enabling professionals to actively participate in designing the improvement processes will provide them with a sense of control of their working conditions that will increase their intrinsic motivation. Institutionalising co-produced quality improvement as organisational routines will show professionals that QI is an ordinary day-today activity that will enrich their clinical practice. These are recommendations that should be paid attention to by healthcare managers who want to further develop QI efforts in their organisations in order to reap the benefits of patient-provider co-production, cited in this article. Healthcare professionals would get an opportunity to improve communication and cooperation with their patients and achieve better outcomes, central to their professional remit. Patients would be able to influence care planning and execution and could offer their expertise in care improvement beyond their own care process.

## Data Availability

The original contributions presented in the study are included in the article/Supplementary Material, further inquiries can be directed to the corresponding author.

## References

[1] Fereday S Rezel K. Patient and Public Involvement in Quality Improvement. Healthcare Quality Improvement Partnership. London: Healthcare Quality Improvement Partnership (2017). Available online at: https://www.hqip.org.uk/wp-content/uploads/2019/05/Nov-2017-update-HQIP-PPI-in-QI-Guide.pdf (Accessed December 15, 2025).

[2] NHS England. Working in Partnership with People and Communities (2022) Available online at: https://www.england.nhs.uk/wp-content/uploads/2023/05/B1762-guidance-on-working-in-partnership-with-people-and-communities-2.pdf (Accessed December 15, 2025).

[3] Iroz CB Godfrey MM Early C Dell B Boyle A Tallarico E A qualitative study to improve how we partner with patients and families in healthcare improvement collaboratives. Health Expect. (2025) 28(3):e70312. 10.1111/hex.70312 40454855 PMC12128466

[4] Gremyr I Elg M Smith F Gustavsson S. Exploring the phase for highest impact on radicality: a cross-sectional study of patient involvement in quality improvement in Swedish healthcare. BMJ Open. (2018) 8(11):e021958. 10.1136/bmjopen-2018-021958 30413500 PMC6231560

[5] Marzban S Marziye N Agolli A Ashrafi E. Impact of patient engagement on healthcare quality: a scoping review. J Patient Exp. (2022) 9:1–12. 10.1177/237437352211254396 PMC9483965 36134145

[6] Bergerum C Engström AK Thor J Wolmesjö M. Patient involvement in quality improvement—a “tug of war” or a dialogue in a learning process to improve healthcare? BMC Health Serv Res. (2020) 20(1):1115. 10.1186/s12913-020-05970-4 33267880 PMC7709309

[7] Bergerum C Wolmesjö M Thor J. Organising and managing patient and public involvement to enhance quality improvement—comparing a Swedish and a Dutch hospital. Health Policy. (2022) 126:603–12. 10.1016/j.healthpol.2022.04.002 35487802

[8] Carstensen K Goldman J Kjeldsen AM Lou S Nielsen CP. Engaging health care professionals in quality improvement: a qualitative study exploring the synergies between projects of professionalisation and institutionalisation in quality improvement collaborative implementation in Denmark. J Health Serv Res Policy. (2024) 29(3):163–72. 10.1177/13558196241231169 38308439 PMC11151708

[9] Vennik FD van de Bovenkamp HM Putters K Grit KJ. Co-production in healthcare: rhetoric and practice. Int Rev. Adm Sci. (2016) 82(1):150–68. 10.1177/0020852315570553

[10] La Grouw Y Ybema S Bannink D. Who empowers who in co-production? Surgeons’ and Patients’ power dynamics throughout clinical decision making. Adm Soc. (2025) 57(4):533–62. 10.1177/00953997251320915

[11] Al-Adili L Roaldseth RJ Pettersen H Brommels M. Inside or outside? A comparative study of two recovery colleges in Sweden and Norway. Submitted January 2026. 10.2196/91076 PMC13460067 42579629

[12] Collins R Shakespeare T Firth L. Psychiatrists’ views on recovery colleges. J Mental Health Training Educ Pract. (2018) 13(2):90–9. 10.1108/jmhtep-05-2017-0037

[13] Grol R Grimshaw J. From best evidence to best practice: effective implementation of change in patients’ care. Lancet. (2003) 362:1225–30. 10.1016/S0140-6736(03)14546-1 14568747

[14] Eccles M Grimshaw J Walker A Johnston M Pitts N. Changing the behavior of healthcare professionals: the use of theory in promoting the uptake of research findings. J Clin Epidemiol. (2005) 58:107–12. 10.1016/j.jclinepi.2004.09.002 15680740

[15] Crano WD Prislin R. Attitudes and persuasion. Annu Rev Psychol. (2006) 57:345–74. 10.1146/annurev.psych.57.102904.190034 16318599

[16] Hagger MS Hamilton K. Progress on theory of planned behavior research: advances in research synthesis and agenda for future research. J Behav Med. (2025) 48(1):43–56. 10.1007/s10865-024-00545-8 39833388 PMC11893630

[17] Medisauskaite A Griffin A Viney R Rashid A Rich A. Changing professional behaviours: mixed methods study utilising psychological theories to evaluate an educational programme for UK medical doctors. BMC Med Educ. (2021) 21(1):92. 10.1186/s12909-021-02510-4 33546673 PMC7866444

[18] Sheeran P. Intention-behavior relations: a conceptual and empirical review. Eur Rev Soc Psychol. (2002) 12:1–36. 10.1080/14792772143000003

[19] Wang D Liu C Zhang X. Do physicians’ attitudes towards patient-centered communication promote physicians’ intention and behavior of involving patients in medical decisions? Int J Environ Res Public Health. (2020) 17(17):1–11. 10.3390/ijerph17176393 PMC7503802 32887364

[20] Wood W. Attitude change: persuasion and social influence. Annu. Rev. Psychol. (2000) 51:539–70. Available online at: www.annualreviews.org (Accessed December 15, 2025). 10751980 10.1146/annurev.psych.51.1.539

[21] Conner M Norman P. Attitudes, intentions, and behavior change. Annu Rev Psychol. (2026) 77(1):311–37. 10.1146/annurev-psych-013125 40825356

[22] Johnson MJ May CR. Promoting professional behaviour change in healthcare: what interventions work, and why? A theory-led overview of systematic reviews. Open. (2015) 5:e008592. 10.1136/bmjopen-2015-008592 PMC4593167 26423853

[23] Potthoff S Kwasnicka D Avery L Finch T Gardner B Hankonen N Changing healthcare professionals’ non-reflective processes to improve the quality of care. Soc Sci Med. (2022) 298:114840. 10.1016/j.socscimed.2022.114840 35287065

[24] Basch E Deal AM Kris MG Scher HI Hudis CA Sabbatini P Symptom monitoring with patient-reported outcomes during routine cancer treatment: a randomized controlled trial. J Clin Oncol. (2016) 34(6):557–65. 10.1200/JCO.2015.63.0830 26644527 PMC4872028

[25] Herman DD Weiss CH Thomson CC. Educational strategies for training in quality improvement and implementation medicine. ATS Scholar. (2020) 1:20–32. 10.34197/ats-scholar.2019-0012PS 33870266 PMC8043285

[26] Johnson JK Batalden P Foster T Batalden M Forcino R Andersson Gare B. A starter’s guide to learning and teaching how to coproduce healthcare services. Int J Qual Health Care. (2021) 33(S2):ii55–62. 10.1093/intqhc/mzab131 34849966

[27] Masterson D Areskoug Josefsson K. Learning from co-creating an online, flexible distance course in co-production in health and welfare. In: Brown J, editor. Abstract Book, International Conference on Work Integrated Learning. Trollhättan, Sweden: University West (2022). p. 57–9.

[28] Kjellström S Sophie Sarre S Masterson D. The complexity of leadership in coproduction practices: a guiding framework based on a systematic literature review. BMC Health Serv Res. (2024) 24:219. 10.1186/s12913-024-10549-4 38368329 PMC10873973

